# Chronic Exposure to the Herbicide, Atrazine, Causes Mitochondrial Dysfunction and Insulin Resistance

**DOI:** 10.1371/journal.pone.0005186

**Published:** 2009-04-13

**Authors:** Soo Lim, Sun Young Ahn, In Chan Song, Myung Hee Chung, Hak Chul Jang, Kyong Soo Park, Ki-Up Lee, Youngmi Kim Pak, Hong Kyu Lee

**Affiliations:** 1 Department of Internal Medicine, Seoul National University College of Medicine, Seoul, Korea; 2 Department of Radiology, Seoul National University College of Medicine, Seoul, Korea; 3 Department of Pharmacology, Seoul National University College of Medicine, Seoul, Korea; 4 Age-Related and Brain Diseases Research Center, Department of Nanopharmaceutical and Life Sciences, Department of Physiology, Kyung Hee University College of Medicine, Seoul, Korea; 5 Department of Internal Medicine, University of Ulsan College of Medicine, Seoul, Korea; Universidad Peruana Cayetano Heredia, Peru

## Abstract

There is an apparent overlap between areas in the USA where the herbicide, atrazine (ATZ), is heavily used and obesity-prevalence maps of people with a BMI over 30. Given that herbicides act on photosystem II of the thylakoid membrane of chloroplasts, which have a functional structure similar to mitochondria, we investigated whether chronic exposure to low concentrations of ATZ might cause obesity or insulin resistance by damaging mitochondrial function. Sprague-Dawley rats (n = 48) were treated for 5 months with low concentrations (30 or 300 µg kg^−1^ day^−1^) of ATZ provided in drinking water. One group of animals was fed a regular diet for the entire period, and another group of animals was fed a high-fat diet (40% fat) for 2 months after 3 months of regular diet. Various parameters of insulin resistance were measured. Morphology and functional activities of mitochondria were evaluated in tissues of ATZ-exposed animals and in isolated mitochondria. Chronic administration of ATZ decreased basal metabolic rate, and increased body weight, intra-abdominal fat and insulin resistance without changing food intake or physical activity level. A high-fat diet further exacerbated insulin resistance and obesity. Mitochondria in skeletal muscle and liver of ATZ-treated rats were swollen with disrupted cristae. ATZ blocked the activities of oxidative phosphorylation complexes I and III, resulting in decreased oxygen consumption. It also suppressed the insulin-mediated phosphorylation of Akt. These results suggest that long-term exposure to the herbicide ATZ might contribute to the development of insulin resistance and obesity, particularly where a high-fat diet is prevalent.

## Introduction

A close association between mitochondrial dysfunction and insulin resistance is well established [1]–[3]. In *in vitro* studies, we found that artificial induction of mitochondrial dysfunction induced insulin resistance [4], [5]. However, there are no *in vivo* studies showing that exposure to an environmental mitochondrial toxin causes insulin resistance.

Persistent organic pollutants (POPs) that contaminate ground and water may accumulate in the tissues of animals and be passed up the food chain, leading to human exposure. Some POPs have recently been associated with the prevalence of diabetes in a serum concentration-dependent manner [6]. The triazine herbicide, atrazine (ATZ, 2-chloro-4-ethylamine-6-isopropylamino-S-triazine), has been extensively used in the USA since the early 1960s, a time frame that corresponds to the beginning of the present obesity epidemic [7], [8]. Because it is moderately persistent under normal soil condition and has low to moderate water-solubility, ATZ is routinely found as a contaminant in many surface and ground waters [9], [10]. Maps of ATZ usage show that the Corn Belt region of the Midwest USA has the heaviest application (http://water.usgs.gov/GIS/browse/herbicide1.gif) (supplementary Figure S1A). Interestingly, the Behavior Risk Factor Surveillance Survey (BRFSS) from 1985 to 2005 by the Center for Disease Control and Prevention revealed a high concentrations of individuals with a body mass index (BMI) over 30 kg/m^2^ in the Corn Belt and surroundings connected via water sources [11] (http://www.cdc.gov/nccdphp/dnpa/obesity/trend/maps/) (supplementary Figure S1B). ATZ-usage and obesity maps show striking overlaps, suggesting that heavy usage of ATZ may be associated with the risk of obesity.

ATZ binds irreversibly to the plastoquinone binding sites of photosystem complex II on thylakoid membranes in chloroplasts, thereby inhibiting electron transport [12]. As mitochondrial electron transfer chain (ETC) complexes I and III also have similar Q binding sites, we hypothesized that ATZ might bind to these mitochondrial sites, resulting in the suppression of mitochondrial oxidative phosphorylation. Previous studies have shown that exposure to ATZ reduces metabolic activity in the gills of fish [13] and induces cellular DNA damage [14]–[18], tumorigenesis [19]–[22], and hermaphroditism of exposed male frogs [23]. In the present study, we found that chronic exposure to low concentrations of ATZ induced abdominal obesity and insulin resistance in rats by impairing mitochondrial function.

## Materials and Methods

### Animals

Male eight-week-old Sprague-Dawley rats (n = 48) were treated for 5 months with vehicle or ATZ (30 or 300 µg kg^−1^ day^−1^) supplied in drinking water. One group of animals was fed a regular diet for the entire period, and another group of animals was fed a high-fat diet (40% fat) for 2 months after 3 months of a regular diet. Initial body weights were the same in both control (187.1±9.4 g) and ATZ (187.5±14.0 g) groups. All rats were fed regular chow (Han Sam R&D, Seoul, Korea) *ad libitum* for three months. Then, half of each group was fed a high-fat diet (high-fat diet group) and the other half was fed regular chow (regular-diet group) for another two months. Regular chow consisted of 16.0% fat, 63.0% carbohydrate and 20.0% protein (by calories), and 7.0% corn oil, 10.0% sucrose, 13.2% dextrose, 40.0% cornstarch, 5.0% cellulose and 20.0% casein (by weight). The high-fat diet consisted of 64.0% fat, 20.0% carbohydrate and 14.0% protein (by calories), and 33.0% shortening, 7.0% corn oil, 10.0% sucrose, 13.2% dextrose, 5.0% cornstarch, 5.0% cellulose and 20.0% casein (by weight). The remaining percentages of the two diets consisted of vitamins and minerals. All rats were maintained in plastic cages in an air-conditioned room at 22±2°C and 55±10% humidity. All procedures were approved by the Institutional Animal Care and Use Committee of the Seoul National University Hospital. The ATZ dose was calculated by multiplying ATZ concentrations by volume of water consumed. Diet and water consumption amounts were measured twice a month.

### Measurement of activity levels

Movement was evaluated using a spontaneous motor activity analyzer (IW-800CT, modular test chamber, Seoul, Korea). Horizontal locomotion and rearing activity were evaluated for 4 hours per month.

### Measurements of obesity

#### 1) Body weight

The rats were weighed twice a month.

#### 2) Visceral fat measurement by high resolution computed tomography (CT)

Visceral fat areas were quantified using non-contrast CT scans (conditions: 120 kVp, 150 mA, 3 mm slice thickness, 3 mm reconstruction interval) using a Somatom Sensation 16 (Siemens, Munich, Germany). With the rats in a supine position, a 3 mm CT slice scan was acquired at the upper margin of the L3 vertebra to measure the amount of abdominal and visceral fat at a single level, and over L1 to L5 for the whole abdomen. Adipose tissue attenuation was determined by measuring the mean value of all pixels within the range of −250 to −50 Hounsfield units. Visceral fat amounts were measured by one radiologist using the computer software, Rapidia (INFINITT, Seoul, Korea).

#### 3) Intramuscular and intrahepatic lipid content by proton magnetic resonance spectroscopy (^1^H-MRS)

Intramuscular lipid (IML) and intrahepatic lipid (IHL) content was measured *in vivo* by ^1^H-MRI, using a 3-Tesla clinical unit (Signa Excite, GE, Milwaukee, WI, USA) with an 8-channel head coil. Voxels of 10 mm^3^ were located in the anterior thigh muscles and liver, avoiding vascular structures and gross adipose tissue deposits. A probe-p sequence (TR/TE = 2,000 ms/35 ms) was used for MRS. Magnetic resonance imaging with the T2-weighted fast spin echo sequences (3,000–3,500/100–120, 18-cm field of view, 256×160 matrix, 3 mm slice thickness) in the axial or coronal planes preceded 1H-MR spectra in order to define the volume of interest. All spectra were processed using Mrdx (CAD Impact, Inc., Seoul, Korea), based on Interactive Data Language (Research Systems, Inc., Boulder, CO, USA). The water signal was suppressed by a frequency-selective saturation pulse at the water resonance value. A sweep width of 5,000 Hz was used with a data size of 2,048 points. Only the second half of the echo was acquired. Following the zero-filling of 8,192 points in all the free induction-decay data, an exponential line-broadening (center 0 ms; half time 150 m) was done before Fourier transformation. Zero-order phase correction was applied to all spectra. The integral of the IML signal (1.3 ppm) was related to that of total creatine (tCr; 3.05 ppm). The IML/tCr ratio corresponded to the total muscle IML value. Fat content was expressed as the ratio of the fat-to-water signal as a percentage. The reliability of the method was assessed by performing repeated measurements on the same individual on different study days and found to be <15%. IML and IHL content was compared between ATZ-treated and normal groups after adjusting for weight.

### Analysis of blood samples for insulin resistance and lipid profiles

Plasma glucose levels were measured using a glucose-oxidase method (YSI 2300-STAT; Yellow Springs Instrument Co., Inc.) immediately after blood was drawn. Serum insulin was measured using insulin-specific radioimmunoassay kits for rats (Linco Research). The homeostasis model assessment-insulin resistance (HOMA-IR) index and beta cell function (HOMA-Beta) were obtained to evaluate insulin resistance and beta cell function. Total cholesterol and triglyceride concentrations were determined by enzymatic procedures (Hitachi 747 chemistry analyzer, Hitachi, Tokyo, Japan).

### Intravenous glucose tolerance test (IVGTT)

IVGTTs were performed as described [24]. All experiments were performed during the light period between 1:00 and 2:00 pm. Animals were food-deprived for 5 hours before starting the experiment. To facilitate stress-free blood sampling, two infusion catheters (PE–10, Intramedic, Clay Adams, Parsippany, NJ, USA) were placed in the tail veins of rats on the evening before the experiment. A bolus dose of 0.5 g glucose/kg body weight was injected into the right tail vein immediately after blood sampling from the left tail vein for measurement of serum concentrations of glucose and insulin (t_0_). Blood samples were collected again from the left tail vein at 2, 4, 6, 10, 20, 30 and 60 minutes for measurement of serum glucose and insulin concentrations. Glucose levels were measured using a YSI 2300 (Yellow Springs Instrument Co., Inc., Yellow Springs, OH, USA). Blood samples were stored at −70°C prior to insulin measurements. Plasma insulin levels were measured using commercial radioimmunoassay kits (Linco Research, St. Charles, MO, USA). Areas under the curve for glucose (AUC_glucose_) and insulin (AUC_insulin_) were calculated using the trapezoid rule for insulin data collected from 0 to 60 minutes.

### Hyperinsulinemic-euglycemic clamp study

Hyperinsulinemic-euglycemic clamping was also performed at the end of the study (5 months) to determine whether ATZ exposure impaired the ability of rats to maintain glucose levels when challenged by a high insulin load, as described [25]. Two tail-vein infusion catheters (PE–10, Intramedic, Clay Adams) were placed in the rats on the evening before the experiment, and one tail-artery blood sampling catheter was emplaced 6 hours before the start of insulin infusion. Whole-body glucose kinetics were estimated in awake, unstressed rats 6 hours after food removal. Patency of the arterial catheter was maintained by a slow infusion (15 µL/minute) of saline. Blood samples (200 µL) were drawn from the tail artery prior to the clamp study to measure baseline fasting glucose and insulin levels. Human insulin (Novolin-R, Novo Nordisk, Bagsvaerd, Denmark) was infused into one of the tail vein microcatheters at a rate of 72 pmol kg^−1^ minute^−1^. Aliquots of venous blood (50 µL) were collected from one tail vein catheter to measure blood glucose at 10-minute intervals, and 25% glucose was infused into the other tail vein catheter at variable rates to maintain plasma glucose at basal concentrations. Steady-state plasma glucose concentrations were reached after 50 to 60 minutes. Blood samples were drawn at 90 and 120 minutes for the measurement of insulin. The steady-state glucose infusion rate (GIR) is defined as the amount of glucose required to maintain euglycemia between 90 and 120 minutes of hyperinsulinemia. The insulin sensitivity index (ISI) was calculated by dividing the GIR by the mean insulin concentration during the 90–120-minute clamping window and expressed as mg glucose kg^−1^ minute^−1^ of for each ng/mL of insulin.

### Energy expenditure

Energy expenditure in rats was measured following a 5-hour fasting period using an Oxymax apparatus (Columbus Instruments, Columbus, OH, USA) two days before each hyperinsulinemic euglycemic clamp experiment [26]. After baseline O_2_, CO_2_ and flow were measured, each rat from each group was situated in metabolic monitor cage for 10 minutes and then O_2_ and CO_2_ were measured again after the values had stabilized. Energy expenditure was calculated according to the formula provided by the manufacturer.

### Electron microscopic images of mitochondria

At the end of the clamp, rats were euthanized with an intravenous injection of pentobarbital, and liver and muscle tissues were collected. The tissue samples were stored frozen at −70°C for later analysis. Soleus muscle and liver tissues were dissected and immersed in fixatives. Mitochondrial morphology was examined by electron microscopy (20,000× magnification).

### Western blot analysis

Cells were washed twice with Dulbecco's phosphate buffered saline (DPBS) and harvested with lysis buffer (50 mM Tris HCl, pH 7.5, 0.1 M NaCl, 1 mM EDTA, 1% Triton X–100, 10 µg/mL each aprotinin and leupeptin, 1 mM PMSF). In some cases, frozen tissue was homogenized in lysis buffer. A portion of cell or tissue lysate (20 µg) was separated by SDS-PAGE on 10–12% gels, transferred to a nitrocellulose membrane (Schleicher & Schnell, Inc., Keene, NH, USA) and analyzed by western blotting using specific antibodies against oxidative phosphorylation (OXPHOS) complex I (39 kDa α subcomplex 9, NDUFA9), complex II (70 kDa flavoprotein, SDHA), complex III (core II, UQCRC2), complex IV-subunit I (COXI, MTCO1), complex IV-subunit IV (COXIV, COX4) or complex V (F1 complex α, ATP5A1). All OXPHOS complex antibodies were purchased from Molecular Probes (Invitrogen, Eugene, OR, USA). The antibodies against Akt and phospho-Akt (Thr308 or Ser473) were purchased from Cell Signaling Technology, Inc (MA, USA). Hsp60 and/or β-actin antibodies were utilized to control for equal loading of protein. The immunoblots were developed using an enhanced chemiluminescence system (ECL, Amersham Pharmacia Biotech., Arlington Heights, IL, USA).

### Endogenous cellular oxygen consumption

Endogenous cellular respiration was measured as described [27], with modifications. Briefly, isolated L6 rat skeletal muscle cells (5×10^6^) in DMEM containing 0.5% FBS were cultured in the presence of ATZ (100 µg/mL) or DMSO (vehicle control) for 48 hours, washed with DPBS (pH 7.4) and collected by trypsinization. After resuspending in 1 mL complete phenol red-free DMEM, the cells were transferred to the chamber of an Oxygraph-2K apparatus (Oroboros, Innsbruck, Austria). Coupled and uncoupled OCRs were measured before and after adding 2.5 µM carbonyl cyanide p-(trifluoromethoxy)phenylhydrazone). KCN-insensitive non-mitochondrial respiration was measured by adding 2.5 mM KCN.

OXPHOS complex activity was measured by placing cells (5×10^6^) in 1 mL of mitochondria respiration buffer (MiRO5, 20 mM HEPES, 110 mM sucrose, 3 mM MgCl_2_, 0.5 mM EGTA, 10 mM KH_2_PO_4_, 60 mM K-lactobionate, 20 mM taurine, 0.1% BSA, pH 7.1 at 30°C) into the continuously stirred chamber of an Oxygraph-2K apparatus (Oroboros, Austria). After recording basal respiration rate, the cells were permeabilized by adding digitonin (25 µg/mL) and adenylate was blocked by adding 100 µM p1,p5-di(adenosine-5′)-pentaphosphate. The activity of each OXPHOS complex was determined by sequential addition of the following inhibitors and substrates: 2 mM ADP, 8 mM malate and 20 mM glutamate for complex I; 1 µM rotenone, 10 mM succinate and 2.5 mM glycerol-3-phosphate for complexes II and III; 25 µM antimycin A, 80 µM ascorbate and 0.42 mM N,N,N,N-tetramethyl-p-phenylenediamine (TMPD) for complex IV; and 2.5 mM KCN for KCN-insensitive respiration. Oxygen consumption rate (OCR) was expressed as picomoles of oxygen consumed per second per milligram of mitochondrial protein.

### Assays for mitochondrial respiratory chain activities

The OCR of complex I, complexes II and III and complex IV of soleus muscle or liver mitochondria were measured using an Oxygraph-2K. Mitochondria were isolated by differential centrifugation, as described previously [28]. Approximately 400 µg mitochondrial protein was suspended in 1 mL MiRO5 buffer. In some cases, isolated mitochondria were incubated with different concentrations (0–300 µg/mL) of ATZ for 30 minutes before OCR measurement. After recording a basal respiration rate, the OCR of each OXPHOS complex was measured by sequential addition of the substrates and inhibitors, as described above.

Because it is not possible to determine the activities of complex II and complex III separately by OCR measurements, the enzyme activities of succinate dehydrogenase (SDH, complex II) and cytochrome *bc*_1_ complex (complex III) of the tissue or cell lysates were assayed spectrophotometrically as described previously [29], with slight modifications. Briefly, tissue or L6 muscle cells were lysed in 100 mM Tris HCl (pH 7.4) containing 250 mM sucrose and 2 mM EDTA. SDH activity was determined by reduction of 2,6-dichlorophenolindophenol (DCPIP) at 600 minus 520 nm (extinction coefficient 19.1 mM/cm) in a mixture of 50 mM potassium phosphate, pH 7.4, 20 mM succinate, 2 µg/mL antimycin A, 2 µg/mL rotenone, 2 mM KCN and 50 µM DCPIP. Complex III activity was measured by reduction of cytochrome *c* at 550 minus 540 nm (extinction coefficient 19.0 mM/cm) in 50 mM Tris-HCl, pH 7.4, 250 mM sucrose, 1 mM EDTA, 0.2 mM KCN, 1 mg/mL antimycin A, 100 µM decylubiquinol and 200 µM oxidized cytochrome *c*. Decylubiquinol was synthesized in the laboratory from decylubiquinone by reduction with potassium borohydride.

Complex I (NADH dehydrogenase) activity was determined in 50 mM potassium phosphate, pH 7.4, 3.5 mg/mL BSA, 0.2 mM NADH, 1 µM antimycin A, 2 mM KCN, 70 µM decylubiquinone and 180 µM DCPIP by measuring the reduction of DCPIP at 520–600 nm (extinction coefficient 19.1 mM/cm) for 1 minute [30]. Complex IV (cytochrome *c* oxidase) activity was determined in 10 mM potassium phosphate (pH 7.4) with 0.1% reduced cytochrome *c* (reduced by sodium hydrosulfate) by measuring oxidation of reduced cytochrome *c* at 550 nm (extinction coefficient 19.0 mM/cm) [29]. Data are expressed as percentages of control activity.

### Statistical analysis

Data are expressed as the mean±SE. Significant differences between groups were evaluated using Student's *t* tests and ANOVA with a post hoc test. Correlations between variables were analyzed using Pearson's correlation coefficient. Differences were considered statistically significant when *P* values were <0.05.

## Results

### ATZ treatments led to weight gain

**Figure 1:**
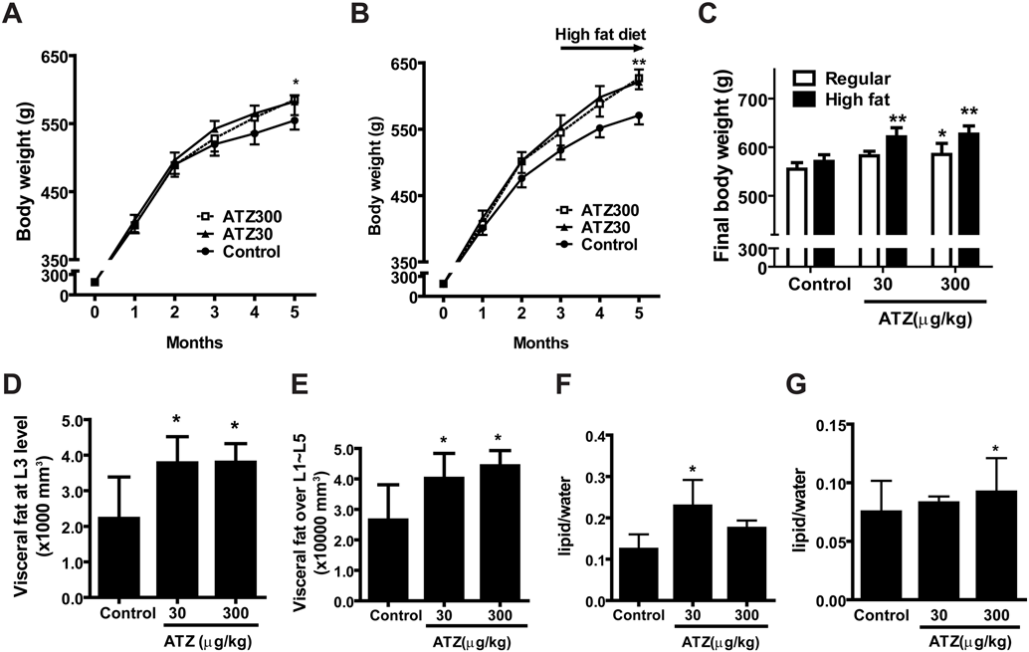
Induction of obesity in rats by ATZ treatment. (A and B) Changes in body weight of ATZ-treated rats (ATZ30, 30 µg kg^−1^ day^−1^; ATZ300, 300 µg kg^−1^ day^−1^) versus control rats over time. (A) Regular-diet group. Rats were fed a regular diet for 5 months during treatment with ATZ, provided in drinking water. (B) High-fat-diet group. Rats were fed a regular diet for 3 months and then fed a high-fat diet for another 2 months. (C) End-of-study comparison of body weights between two different diet-treated rats. (D and E) Increase in visceral fat by ATZ. The amount of visceral fat in the high-fat diet group was measured by horizontal CT scan. Abdominal fat area at the L3 level (D) and over L1 to L5 (E) were calculated from the scanned image using a Hounsfield unit. (E and F) Intracellular fat deposition by ATZ. The amount of intrahepatic (F) and intramuscular (G) fat in rats on a regular diet was measured by non-invasive ^1^H-MRS and adjusted for body weight. (*p<0.05, **p<0.01 vs. control).

After being fed a normal diet for 3 months, control and the ATZ-treated rats exhibited no significant differences in mean body weight. From month 4 onward, mean body weight in the regular-diet groups was higher for ATZ-treated rats than for control rats. At the end of the study, the body weights of the regular-diet rats were 582.3±9.3 g for the 30-µg kg^−1^ day^−1^ ATZ group (ATZ30), 585.0±22.7 g for the 300-µg kg^−1^ day^−1^ ATZ group (ATZ300) and 554.6±13.4 g for controls; overall, the mean body weight of ATZ-treated rats was 5.5% higher than that of the controls (p<0.05; **Fig. 1A and 1C**). In the high-fat diet groups, the body weights of ATZ30 and ATZ300 groups were 621.1±18.8 g and 626.9±16.9 g, respectively, or 9.8% higher overall than control rats (570.8±13.6 g) (p<0.01, **Fig. 1B and 1C**). No treatment-related differences in food/water intake or horizontal/spontaneous locomotor activities were observed at any point. Thus, chronic exposure to low concentrations of ATZ resulted in weight gain, particularly when combined with a high-fat diet.

### ATZ increased visceral fat and intracellular fat content

CT scans were utilized to determine whether the weight gain induced by ATZ was related to the amount of visceral fat. In high-fat diet groups, ATZ-treated rats had a greater amount of visceral fat at the L3 level and between L1 and L5 than controls (p<0.05; n = 8/group; **Fig. 1D and 1E**). Fat deposition in the muscle and liver is positively correlated with measures of obesity and negatively with insulin sensitivity [24]. Non-invasive ^1^H-MRS revealed that, even in regular diet groups, ATZ treatment increased intramuscular and intrahepatic fat accumulation, with borderline significance after adjusting for body weight (**Fig. 1F and 1G**). Thus, ATZ treatment for 5 months enhanced visceral fat accumulation in the groups fed a high-fat diet and induced lipid accumulation in both muscle and liver in rats fed a regular diet.

### ATZ treatment worsened insulin levels and HOMA-IR values

For rats fed a regular diet, ATZ treatment tended to increase triglyceride and total cholesterol levels, but the differences did not reach statistical significance (data not shown). However, ATZ accelerated the increase in triglycerides induced by the high-fat diet (p<0.05 vs. control), and also worsened insulin levels and HOMA-IR index values. Specifically, in high-fat diet groups, insulin levels were increased to 2.39±0.91 and 2.21±0.86 ng/mL in the ATZ300 and ATZ30 groups, respectively compared to 1.70±0.57 ng/mL in controls (p<0.01). The corresponding HOMA-IR values for ATZ300, ATZ30, and control groups were 0.60±0.24, 0.51±0.20 and 0.37±0.11, respectively (p<0.05).

### ATZ induced insulin resistance

**Figure 2:**
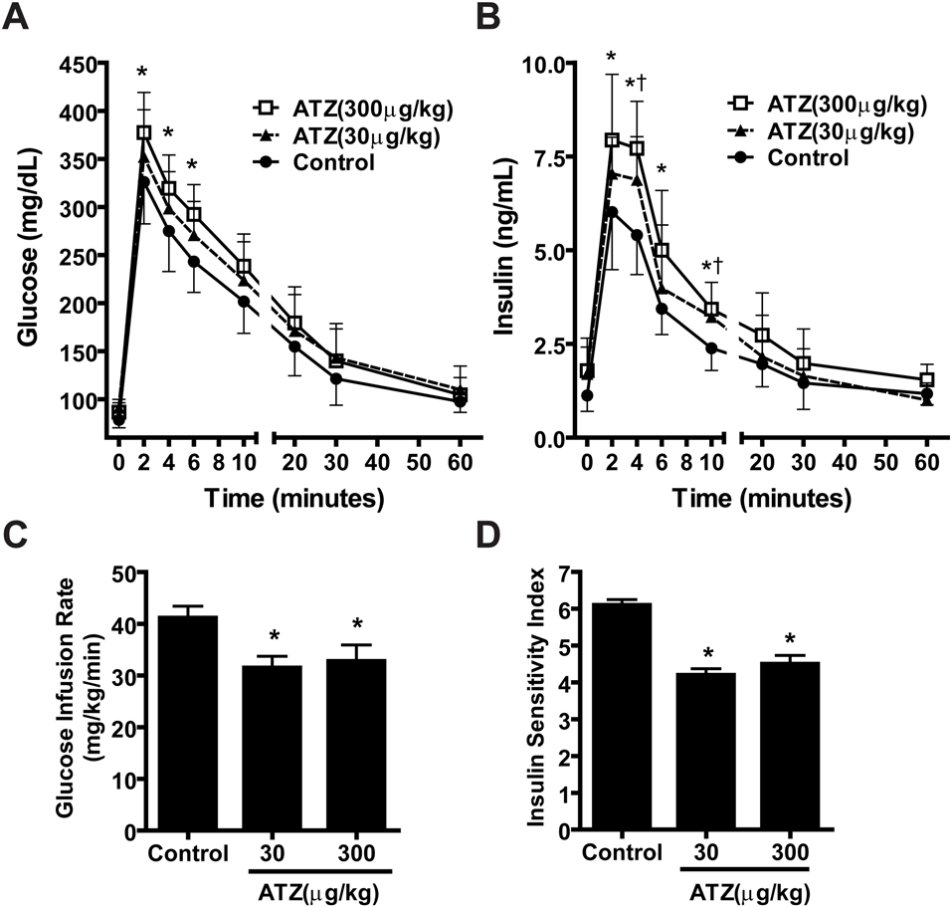
Impairment of IVGTT and insulin sensitivity by ATZ exposure in regular-diet rats. (A) Changes in plasma glucose during IVGTT. (B) Changes in plasma insulin during IVGTT. Plasma glucose or insulin concentrations were determined at the indicated times after i.v. glucose load. (C) Steady-state glucose infusion rate (GIR) and (D) insulin sensitivity index (ISI) during clamp study. (*p<0.05 vs. control; n = 8 per group).

To determine if ATZ treatment induced glucose intolerance and insulin resistance, we performed IVGTTs and a hyperinsulinemic-euglycemic clamp study. Fasting plasma glucose levels of ATZ-treated rats on the regular diet were higher than those in controls (86.5±9.8, 91.1±8.5, and 78.5±8.3 mg/dL in ATZ30, ATZ300 and controls, respectively; p<0.05), although they were within the normal range. The same pattern was found in the high-fat diet group: fasting glucose levels were 97.8±6.5 and 102±8.3 in ATZ30 and ATZ300 groups, respectively, compared to 90.0±6.7 mg/dL in controls (p<0.05). Interestingly, ATZ exposure impaired glucose tolerance as monitored by IVGTT. Plasma glucose and insulin concentrations in both ATZ-treated groups were significantly higher than those in controls at early points (<10 min) after intravenous loads of glucose (p<0.05 vs. control) (**Fig. 2A and 2B**).

The hyperinsulinemic-euglycemic clamp study demonstrates an animal's ability to maintain glucose levels when challenged by a high insulin load. Steady-state plasma insulin concentrations were measured as plasma glucose reached steady state during clamping. For the ATZ-treated rats on a regular diet, insulin levels increased to 7.20±0.70 ng/mL in ATZ30 and 7.50±0.50 ng/mL in ATZ300 compared with 4.90±0.60 ng/mL glucose infusion rate for controls (p<0.05 for both). AZT significantly decreased the steady-state glucose infusion rate (GIR) during the clamp to 31.5±2.22 and 32.75±3.18 mg kg^−1^ minute^−1^ for ATZ30 and ATZ300, respectively compared to 41.13±2.28 mg kg^−1^ minute^−1^ for controls (p<0.05 for both) (**Fig. 2C**). Thus, the ATZ-exposed rats showed an impaired response to insulin loading, even under a regular diet. The insulin sensitivity index (ISI) was also significantly reduced to 4.2±0.17 for ATZ30 and 4.5±0.23 for ATZ300 compared with 6.1±0.15 for controls (p<0.05 for both; **Fig. 2D**). Collectively, these data support the conclusion that rats chronically exposed to ATZ developed insulin resistance without being fed a high-fat diet.

### ATZ changed the ultrastructure of mitochondria

**Figure 3:**
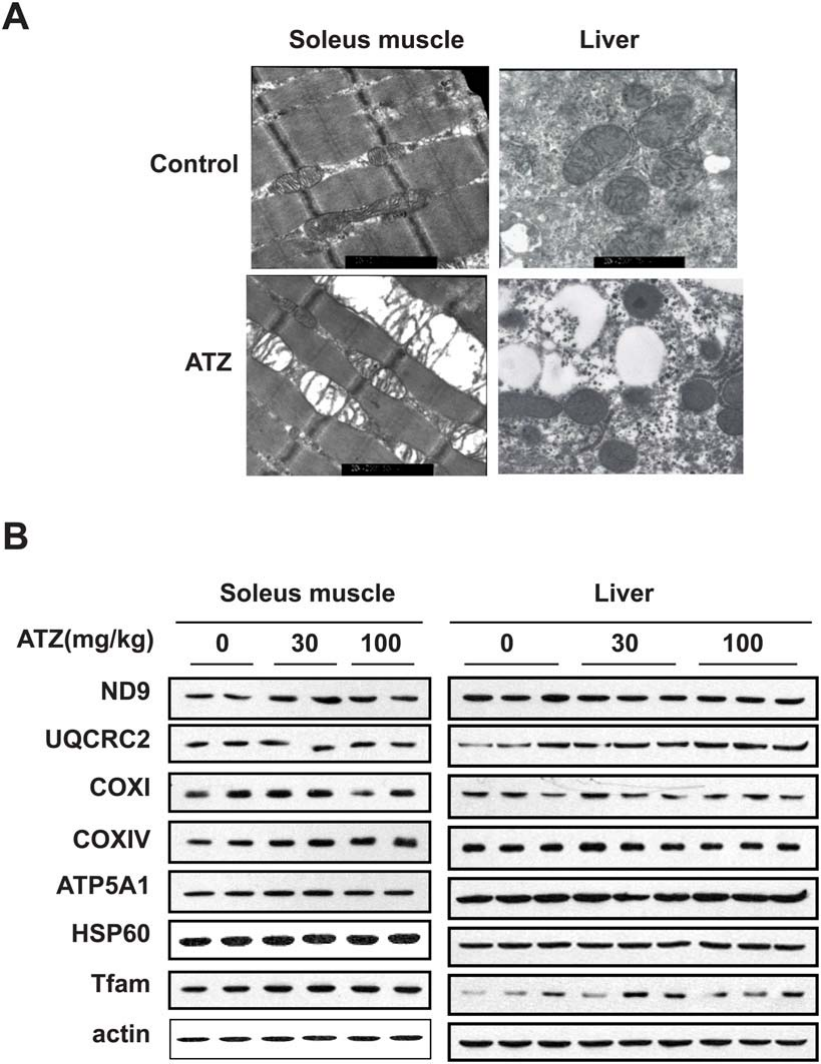
Mitochondrial morphology and protein expression in ATZ-treated rat muscle and liver. (A) Mitochondrial morphology by electron microscopy. Rats on a regular diet were treated with or without ATZ300 for 5 months. Rat soleus muscle and liver were isolated and observed by electron microscopy (magnification ×20,000). Enlarged, swollen or cristae-disrupted mitochondria are indicated by arrows. Lipid droplets were observed in ATZ-treated liver. (B) Expression of mitochondrial proteins in rat tissues. Total lysates of soleus muscle or liver of rats on a regular diet with or without ATZ treatment were analyzed by western blotting using the indicated antibodies.

The soleus muscles of ATZ-treated rats were not discernibly different from those of control rats by light microscopy. However, electron microscopy showed that soleus muscle mitochondria in these animals on a regular diet were swollen and their cristae were partially destroyed (**Fig. 3A**). There was also prominent accumulation of lipid droplets in the livers of ATZ-treated rats, and electron microscopy revealed that some liver mitochondria from the ATZ-treated group showed partially disrupted cristae. Despite the fact that mitochondrial morphology was altered in muscle and liver by ATZ administration, protein expression levels of mitochondrial OXPHOS complex subunits in liver and muscle tissues were not changed significantly (**Fig. 3B**). Therefore, the AZT-induced disruption of mitochondrial morphology might not be associated with changes in mitochondrial complex protein expression.

### ATZ exposure decreased energy metabolism

**Figure 4:**
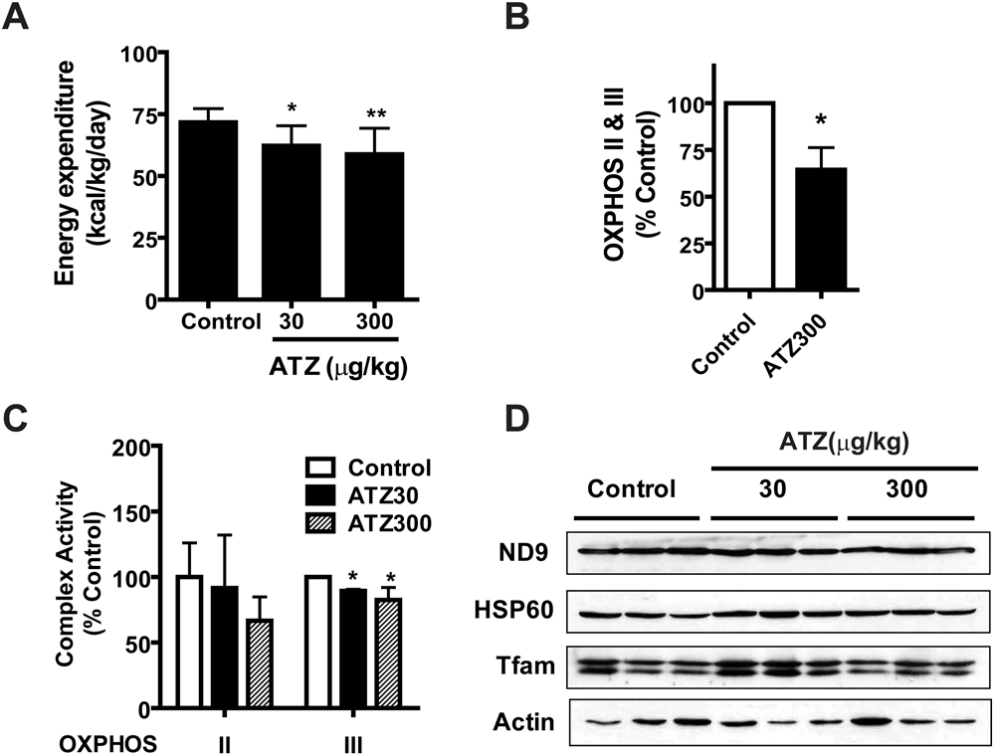
Mitochondrial OXPHOS activity of ATZ-treated rat skeletal muscle mitochondria. Rats on a regular diet were treated with or without ATZ for 5 months. (A) Dose-dependent decrease in energy expenditure in ATZ-treated rats, monitored using indirect calorimetry. (B) Decrease in OCR of complex II plus III in ATZ300-treated skeletal muscle mitochondria (n = 5). (C) Decrease in the activity of complex III enzyme in ATZ-treated liver lysates, determined by spectrophotometry (*p<0.05, **p<0.01; n = 5). (D) Western blot of muscle mitochondria proteins. Total skeletal muscle mitochondrial lysates from rats on a regular diet were subjected to western blot analysis using the indicated antibodies.

Insulin resistance and obesity are affected by caloric intake, physical activity and energy expenditure [31], [32]. Since no treatment-related changes in food or water intake or physical activity were observed at any point during the study, the development of insulin resistance by ATZ might be related to energy metabolism. To determine if ATZ affected energy metabolism, we measured energy expenditure and OCR using indirect calorimetry and oxygen sensors, respectively. The energy expenditure of ATZ-treated rats on regular diet was reduced in a dose-dependent manner (**Fig. 4A**). The OCR of each OXPHOS complex was measured using mitochondria isolated from the soleus muscle of ATZ-treated rats on regular diet. The combined OCR measured for complexes II plus III, both of which use ubiquinone (Q) as an electron transfer intermediate, was significantly reduced by ATZ administration. The activities of other complexes were not altered (**Fig. 4B**), although the OCR of complex I showed a tendency toward a decrease that did not reach statistical significance. There were some analytical limitations in measuring OCR where the changes were modest, especially when using animal tissues.

To distinguish which of these complexes (II or III) were affected by ATZ, we measured the enzymatic activities of SDH (complex II) and cytochrome bc_1_ (complex III) in gastrocnemius muscle tissues by spectrophotometry. As expected, SDH activity was not altered by ATZ treatment while the activity of the cytochrome *bc*_1_ complex was decreased by 10% in the muscles of ATZ-treated animals (p<0.05, **Fig. 4C**). These results suggest that ATZ may bind to the Q site of the cytochrome *bc*_1_ complex, a counterpart of the plastoquinone of photosynthesis in plants. Consistent with tissue lysate western blotting results, the amounts of OXPHOS complex proteins in mitochondria were not significantly altered. The western blot results imply that ATZ might interfere with the electron transfer from complex I or II to complex III without impacting mitochondrial OXPHOS protein expression levels (**Fig. 4D**). However, we cannot exclude the possibility that the expression of other proteins that were not examined by western blotting might be involved in changes in mitochondria respiration activity.

### ATZ impairs mitochondrial OXHPOS through direct action

**Figure 5:**
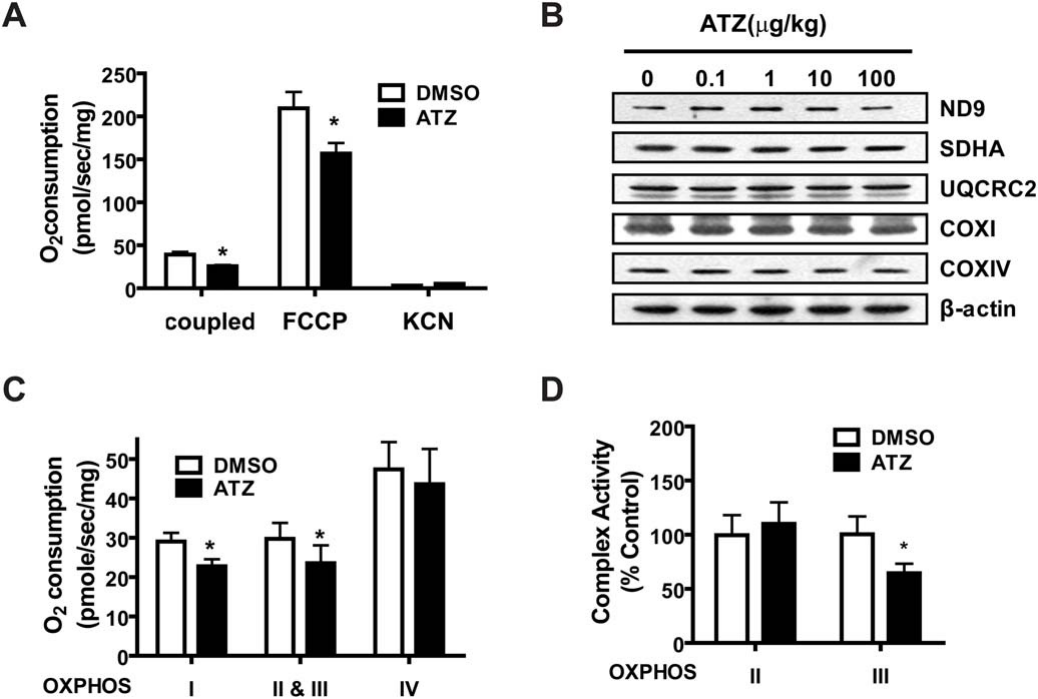
Decrease in endogenous oxygen consumption by ATZ in L6 cells. L6 rat skeletal muscle cells were treated for 48 hours with or without ATZ (100 µg/mL). (A) Endogenous cellular (coupled), FCCP-uncoupled (FCCP) and KCN-insensitive respiration (KCN) of trypsinized intact cells were measured in phenol red-free media using an Oxygraph-2K apparatus (*P<0.05; n = 3). (B) Western analysis of nDNA- and mtDNA-encoded OXPHOS complex subunit proteins. Complex I (ND9), complex II (SDHA), complex III (UQCRC2) and complex IV (COXI, mtDNA-encoded, COXIV, nDNA-encoded) were examined. β-actin was used as an equal loading control. (C) Oxygen consumption by each complex in digitonin-permeabilized cells was measured using an Oxygraph-2K apparatus (*P*<0.05; n = 4). (D) Enzyme activities of complex II or III were determined by spectrophotometry in ATZ-treated L6 muscle cells (*P<0.05; n = 3).

**Figure 6:**
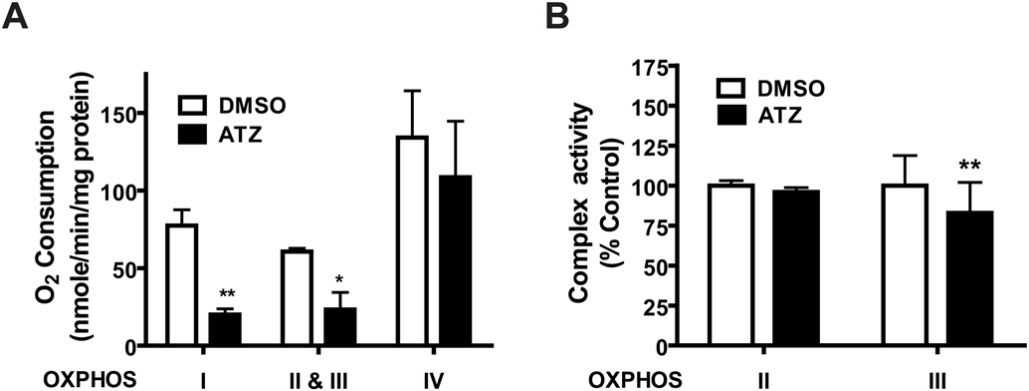
Inhibition of complex I and II plus III in isolated mitochondria by ATZ. (A) OCR. Mouse liver mitochondria (40 µg/100 µL assay) isolated by differential centrifugation were incubated with ATZ (100 µg/mL) for 30 minutes and the OCR of each complex was measured. (B) Enzyme activities of complex II (SDH) or complex III (cytochrome *bc*1 complex). Liver tissue lysates of ATZ-treated mitochondria were assayed as described (*p<0.05, **p<0.01; n = 4).

Changes in cellular respiration and differences in the respiratory effects of inhibitors are important indicators of mitochondrial functional defects that result from damaged mitochondrial proteins or DNA (mtDNA), or substantial alterations to mitochondrial signaling cascades. To test if ATZ impaired mitochondrial function directly or indirectly, we monitored both cellular and mitochondrial respiration levels in ATZ-treated, cultured L6 muscle cells and isolated mitochondria. Incubation of L6 muscle cells with ATZ decreased both endogenous coupled and FCCP-uncoupled oxygen consumption by 30% and 40%, respectively (**Fig. 5A**). ATZ had no effect on the levels of nuclear DNA (nDNA)- or mtDNA-encoded OXPHOS complex proteins (**Fig. 5B**). When oxygen consumption was measured using digitonin-permeabilized cells, respiration levels in complex II plus III were inhibited by ATZ (**Fig. 5C**), similar to the *in vivo* results (**see** **Fig. 4C**). Enzyme activities of complex III (cytochrome *bc*_1_) were also decreased by ATZ treatment (**Fig. 5D**). Direct treatment of isolated mouse liver mitochondria with ATZ also reduced the OCR of complex I and complex II plus III by 49% and 37%, respectively (**Fig. 6A**); the magnitude of these ATZ effects were greater in isolated mitochondria than in cells. Complex IV activities were not significantly changed. Again, consistent with the results of the *in vivo* study, SDH enzymatic activity was not changed, but the activity of the cytochrome *bc*_1_ complex was decreased significantly by ATZ treatment (**Fig. 6B**). These results strongly suggest that ATZ itself, not ATZ metabolites, intervenes directly at Q binding sites between complex I and III, or II and III.

### ATZ treatment blocked the insulin-Akt signaling pathway

**Figure 7:**
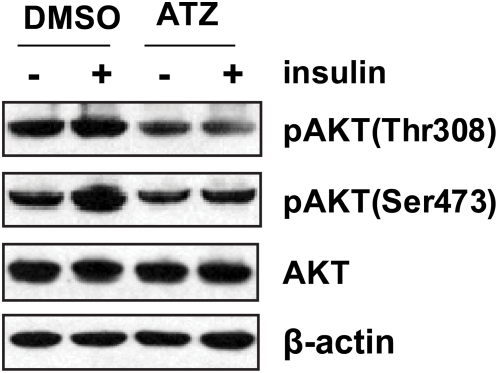
Inhibition of insulin signaling by ATZ. L6 cells were treated with ATZ (100 µg/mL) in DMEM containing 0.5% FBS for 24 hours and stimulated with insulin (100 ng/mL) for 30 minutes. The cells were harvested and analyzed by western blotting using anti-Akt or -pAkt antibodies. Akt Thr308 and Ser473 phosphorylation, both important in the insulin-signaling pathway, were blocked by ATZ treatment.

To investigate the mechanism by which ATZ-induced mitochondrial dysfunction caused insulin resistance, we analyzed insulin-stimulated Akt phosphorylation in ATZ-treated L6 muscle cells. Pretreatment with ATZ (100 µg/mL) for 24–48 hours abolished insulin-mediated Akt phosphorylation at both Thr308 and Ser473 residues (**Fig. 7**). Park *et al.* demonstrated that mitochondrial dysfunction induced by mtDNA depletion suppressed IRS-1 expression, resulting in diminished downstream signaling and glucose transport, and insulin resistance [5]. The suppression of insulin-mediated Akt phosphorylation is clearly linked to the development of insulin resistance *in vitro* [33] and *in vivo* [34]. Thus, the reduction in Akt phosphorylation that results from ATZ-mediated inhibition of mitochondrial OXPHOS complex III may help to explain why chronic exposure to ATZ induced weight gain and insulin resistance.

## Discussion

In this study, we found that long-term treatment with low concentrations of the herbicide, ATZ, induced insulin resistance and weight gain in Sprague Dawley rats. We acknowledge that, unlike the current results, previous studies have reported that the body weights of ATZ-treated animals were generally decreased or unchanged [35]–[37]. However, the doses of ATZ used in these other studies were 10–100 fold higher (2.7 to 50 mg kg^−1^ day^−1^) than those used in our study. Thus, our interpretation is that acute exposure to high concentrations of ATZ is toxic, and thus prevents weight gain and possibly causes weight loss. In contrast, chronic low-dose ATZ exposure might lead to mild mitochondrial damage that mimics the characteristic of the insulin-resistance state, and hence, leads to weight gain.

Because ATZ treatment in our study induced obesity without changing food intake or physical activity, ATZ presumably lowered energy metabolism. Indeed, indirect calorimetry measurements revealed that the ATZ-induced weight gain was associated with decreased energy metabolism. Furthermore, *in vitro* experiments provided evidence that ATZ interferes with electron transfer through OXPHOS complex at Q sites in mitochondria, resulting in reduced oxygen consumption. We have found that treatment with rotenone, a complex I inhibitor, or dideoxydytidine, an mtDNA-replication inhibitor, directly inhibits insulin-mediated phosphorylation of Akt without affecting upstream signaling molecules (data not shown). In addition, overexpression of dominant-positive Akt (myr-Akt) completely reverses downstream Akt function. For example, Akt-mediated phosphorylation of Foxo1, which is blocked by mitochondrial dysfunction, is abrogated by myr-Akt overexpression, implying that Akt is the focal point of the mitochondrial dysfunction (manuscript in preparation). As part of this study, we tested whether ATZ also blocked Akt function, and found, as expected, that ATZ blocked insulin-mediated Akt phosphorylation in skeletal muscle cells.

Treatment with ATZ changed the ultrastructure of mitochondria in liver and muscle of rats, producing morphological alterations such as ring- and cup-shaped mitochondria without significantly altering the expression of mitochondrial OXPHOS complex proteins. This observation is in agreement with the findings of others that ATZ induced mitochondrial damage in fresh water mussels [13] and rainbow trout [38]. In our study, we also found that ATZ decreased the membrane potential of mitochondria and reduced intracellular ATP content in various cells (data not shown). Collectively, these data led us to conclude that ATZ treatment damaged both mitochondrial respiratory function and morphology.

Intramuscular lipid accumulation is known to be closely related to insulin resistance [39], [40], and is considered as an early phenomenon in the development of obesity. Increased lipid content in muscle is also associated with decreased ATP synthesis and diminished mitochondrial function [1]. The observed increase in intracellular lipid content in ATZ-treated rats also supports the interpretation that ATZ-induced mitochondrial damage affects the insulin-signaling pathway, and consequently induces insulin resistance and fat accumulation in metabolically active tissue, such as muscle.

There is epidemiological evidence that human exposure to POPs such as TCDD may also disturb glucose metabolism and induce insulin resistance [6], [41]–[47], and it has been reported that exposure to herbicides or pesticides, including ATZ, is associated with an increased risk of gestational diabetes [47]. However, there is scant information in the literature to indicate the level of human exposure to ATZ or similar herbicides. Obviously, there is no direct evidence of ATZ accumulation in diabetic or obese human subjects. However, a toxicological report has shown that acute occupational or dietary exposure of humans to ATZ was in the range of 0.2–90 µg kg^−1^ day^−1^, and the annual average exposure (chronic) was between 0.046 and 0.286 µg kg^−1^ day^−1^ [48].

We believe that ATZ or its metabolites may be introduced to humans through air, water and and/or corn products as contaminants, and accumulate in tissues. One such pathway by which ATZ or its metabolites might be introduced into humans is through corn-derived foods (e.g., high fructose corn syrup or corn oil). Since corn syrup [49] and fast foods served in the USA [50], [51] are suspected of causing an obesity epidemic, this seems a reasonable supposition. Recently, it was reported that of 160 food products purchased at a fast food restaurant throughout the USA, not a single item could be traced back to a non-corn source [52]. This work also identified corn as the overwhelmingly predominant animal feed for the beef and chicken served at fast food restaurants.

Considering that the process of corn wet milling requires a huge amount of fresh water for steeping (digesting), which in turn generates 1,300 to 1,600 L of light steepwater per ton of corn that is then evaporated or dried to make intermediate products, it is conceivable that ATZ and related herbicides are present in steepwater and may be concentrated during the process [53]. Because herbicides like ATZ and their metabolites are present in streams and groundwaters throughout USA and are at highest concentrations in agricultural areas where wet corn milling plants are located [54], there is a substantial possibility of ATZ contamination of corn products. However, in 2006 the U.S. Environmental Protection Agency issued a cumulative risk assessment of triazine herbicides, concluding that they posed “no harm that would result to the general U.S. population, infants, children, or other consumers” [55].

Based on evidence presented in this study, we conclude that environmental ATZ might be an important contributing factor to the obesity epidemic in the United States. It damages mitochondrial function, affects insulin signaling, and induces insulin resistance and obesity, especially when exposure is associated with a high-fat diet. Sanguine official assessments notwithstanding, further studies are definitely needed to clarify issues related to human exposure to ATZ and related herbicides in the US and elsewhere around the world.

## Supporting Information

Figure S1 Comparison of atrazine usage map and obesity trend in U. S. A.
(2.51 MB TIF)
