# Supplementary figures and images for: Chronic Exposure to the Herbicide, Atrazine, Causes Mitochondrial Dysfunction and Insulin Resistance
Source: PLoS One. 2009 Apr 13;4(4):e5186. doi: 10.1371/journal.pone.0005186 (PMC2664469; doi:10.1371/journal.pone.0005186)

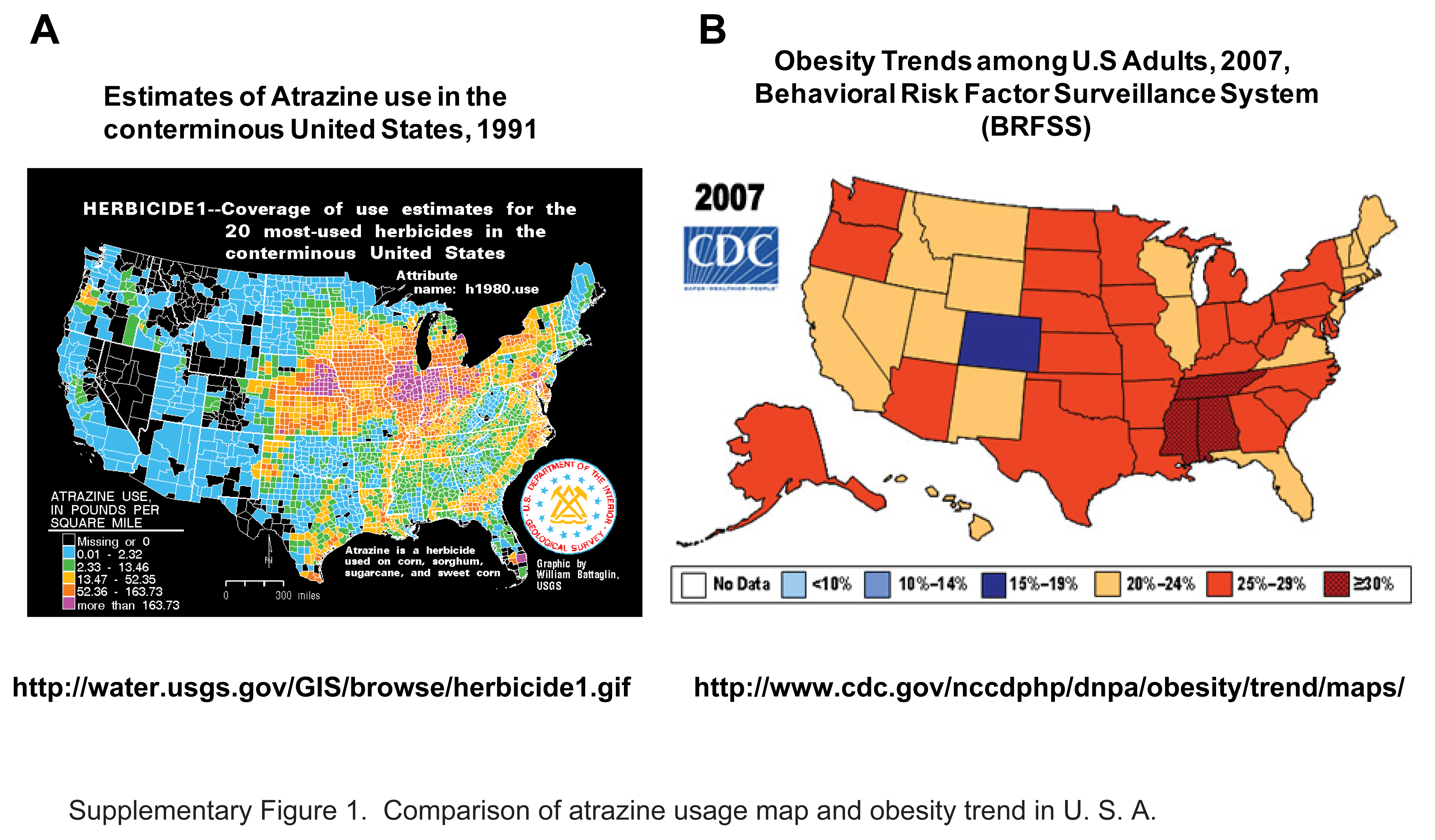

Supplement: Figure S1 — Comparison of atrazine usage map and obesity trend in U. S. A. (2.51 MB TIF) [file pone.0005186.s001.tif]
